# What Makes a Successful Partnership? Lessons from a Multi-Agency Equity-Focused Maternal and Early Childhood Health Partnership in Victoria, Australia: A Case Study

**DOI:** 10.1177/00469580251336098

**Published:** 2025-05-27

**Authors:** L. J. Biggs, F. Gordon, J. Leetham, K. Pangli Kaur, F. Hearn, G. Callandar, J. Szwarc, H. Brundell, K. Diacono, E. Riggs, S. J. Brown

**Affiliations:** 1 Murdoch Children’s Research Institute (MCRI), Parkville, Vic, Australia; 2 Western Health, Bacchus Marsh, Vic, Australia; 3 Melton City Council Maternal and Child Health Service, Melton, Vic, Australia; 4 VICSEG New Futures, Coburg, Vic, Australia; 5 Victorian Foundation for Survivors of Torture (Foundation House), Brunswick, Vic, Australia; 6 University of Melbourne, Parkville, Vic, Australia; 7 South Australian Health and Medical Research Institute (SAHMRI), Adelaide, SA, Australia

**Keywords:** health equity, pregnancy, early childhood, social determinants of health, multi-agency partnership, multi-disciplinary partnership, evaluation

## Abstract

Disparities in maternal and child health outcomes in high-income countries, including Australia, are influenced by complex social determinants of health. We formed a multi-disciplinary multi-agency partnership to improve the capacity of maternity and early childhood health workforces to identify and respond to social factors contributing to poorer perinatal outcomes for families in a Local Government Area in Victoria, Australia. This case study reports our experiences and reflections on working in partnership during the COVID-19 pandemic. An evaluation involving semi-structured interviews and a collaborative workshop attended by 10 partnership group members was undertaken in 2023. Recognising that the pandemic had constrained the implementation of many project activities, the aims of the evaluation were: to understand what had worked well within the partnership group and why, and to identify insights and recommendations to inform government and policy makers, health service managers, and teams seeking to undertake similar work. Partnership members valued the opportunity and time invested in building and maintaining relationships across agencies and between team members. Disruption to services associated with the COVID-19 pandemic amplified established cultural, political, and organisational barriers to service innovation and health equity. Despite the challenges of undertaking this work during the COVID-19 pandemic, our positive collaborative relationships and shared sense of purpose have supported us to remain hopeful about the potential for future health equity initiatives to achieve change. Reflecting our optimism, we have co-produced a set of recommendations for teams seeking to undertake similar work in the future.

**What do we already know about this topic?**
Multi-disciplinary multi-agency partnerships can create the conditions necessary for the system and practice innovation integral to addressing health inequities.
**How does your research contribute to field?**
Our experiences of working in partnership highlight the importance of taking the time to establish and maintain connections across organisations and between team members is critical to enable innovation.
**What are your research’s implications toward theory, practice, or policy?**
Insight from our evaluation, including a set of co-produced recommendations, can be used to inform future health equity innovations and health and social care partnerships more broadly.

## Introduction

In Australia, as in other high-income countries, there are stark disparities in perinatal outcomes. Low birthweight, preterm birth and stillbirth are more common for Aboriginal and Torres Strait Islander and some refugee and migrant background families, women who experience intimate partner violence and/or other social health issues during pregnancy, and families residing in ‘deprived’ neighbourhoods.^1-10^ Several major international reports have identified investment in strategies to promote ‘a healthy start to life’ as having the greatest potential to reduce health inequalities across the life course.^7,10,11^ Despite this, progress to address disparities in perinatal outcomes is limited, and there are grounds for concern that disparities are increasing in some practice settings.^12-14^

Multi-agency multi-disciplinary partnerships (groups with members from different organisations, professional and disciplinary backgrounds) have been identified as a useful strategy for creating the conditions necessary for the system and practice innovation integral to addressing health inequities.^15-17^ Despite their importance, little has been published regarding approaches to forming and sustaining such partnerships, particularly during time of uncertainty, such as the COVID-19 pandemic.^18-20^ Context-specific case studies from teams designing and implementing interventions to reduce health inequities have the potential to offer insight into what works to establish and maintain partnerships in dynamic health and social care settings characterised by uncertainty and unpredictability.^21,22^

We report insights from a 2-year multi-agency multi-disciplinary partnership established in the second year of the COVID-19 pandemic, which aimed to reduce perinatal disparities for women, babies, and families of refugee and migrant backgrounds in a Local Government Area of Melbourne, Australia. Despite multiple challenges posed by pandemic restrictions and other pressures, the *Strong Families Strong Babies* partnership group sustained connection and shared purpose over a 2-year implementation period. This case study focuses on our experiences of collaborative partnership within this project, with a view to informing the work of other teams seeking to establish and sustain equity-focused multi-agency partnerships.

### The Strong Families Strong Babies Project

*Strong Families Strong Babies* sought to co-design, implement, and evaluate a number of organisational and practice changes to re-orient maternity and early childhood healthcare to help address the social determinants of health known to contribute to perinatal disparities. The project was commissioned by the North Western Melbourne Primary Health Network with a view to improving birth outcomes for families living in the City of Melton Local Government Area of Melbourne, Victoria Australia and specifically in the Melton South region. The suburb of Melton is located 37 km west of Melbourne’s Central Business District, with a population of 178 960 people with a median age of 33.^23^ The area is amongst the most socioeconomically disadvantaged suburbs in the state of Victoria, and known to have a higher proportion of infants born with a low birthweight and children identified as developmentally vulnerable on the Australian Early Development Index compared with more socio-economically advantaged localities. The area also has a large number of families with refugee or migrant backgrounds. Through community and stakeholder engagement, the partnership group aimed to identify and co-design equity-focused practice change initiatives which, if successful, would improve maternity and early childhood healthcare providers’ ability to address modifiable social risk factors contributing to disparities in maternal and child health outcomes.

## Partnership Group

The project was undertaken as a partnership between the Intergenerational Health group at the Murdoch Children’s Research Institute, Western Health Bacchus Marsh campus, Melton City Council Maternal and Child Health (MCH) Service, VICSEG New Futures, and the Victorian Foundation for Survivors of Torture (Foundation House; Table 1). A partnership group was established with representatives from each agency in early 2021 to oversee co-design, implementation, and evaluation of the project. The group included diverse skills and experience in clinical practice, management, policy, and research, recognised in past research as important for partnership success.^24^

**Table 1.** Partner Agencies and Responsibilities.

Agency	Responsibility
Murdoch Children’s Research Institute	• Partnership group facilitation
	• Research conduct, governance, and ethics
	• Maternity and early childhood, health services, and health equity practice innovation expertise
Melton City Council Maternal and Child Health Service	• Partnership group participation
	• Contribution of maternal and child health expertise
	• Knowledge of local communities and workforces
	• Maternal and child health service delivery
Western Health Bacchus Marsh (Djerriwarrh Health Services until July 2021)	• Partnership group participation
	• Maternity and community paediatric expertise
	• Knowledge of local communities and workforces
	• Maternity (midwifery) service delivery
VICSEG New Futures	• Partnership group participation
	• Community-based refugee and migrant background support service expertise
	• Bicultural mentor service delivery
Victorian Foundation for Survivors of Torture	• Partnership group participation
	• Community-based refugee trauma support service expertise
	• Professional development for health professionals at partner agencies

Western Health is the main provider of public maternity services in the area, with approximately 550 births at the Bacchus Marsh campus per year. The Melton MCH Service provides key age-related health and developmental assessments and enhanced support services to families across the City of Melton, including families living in Melton South. VICSEG New Futures is a not-for-profit organisation that uses skill building and a focus on social equity to work with refugees, those seeking asylum, and migrants. Foundation House provides counselling services and advocacy to support people of refugee background to rebuild their lives after experiences of torture or other traumatic events.

**Figure 1.**
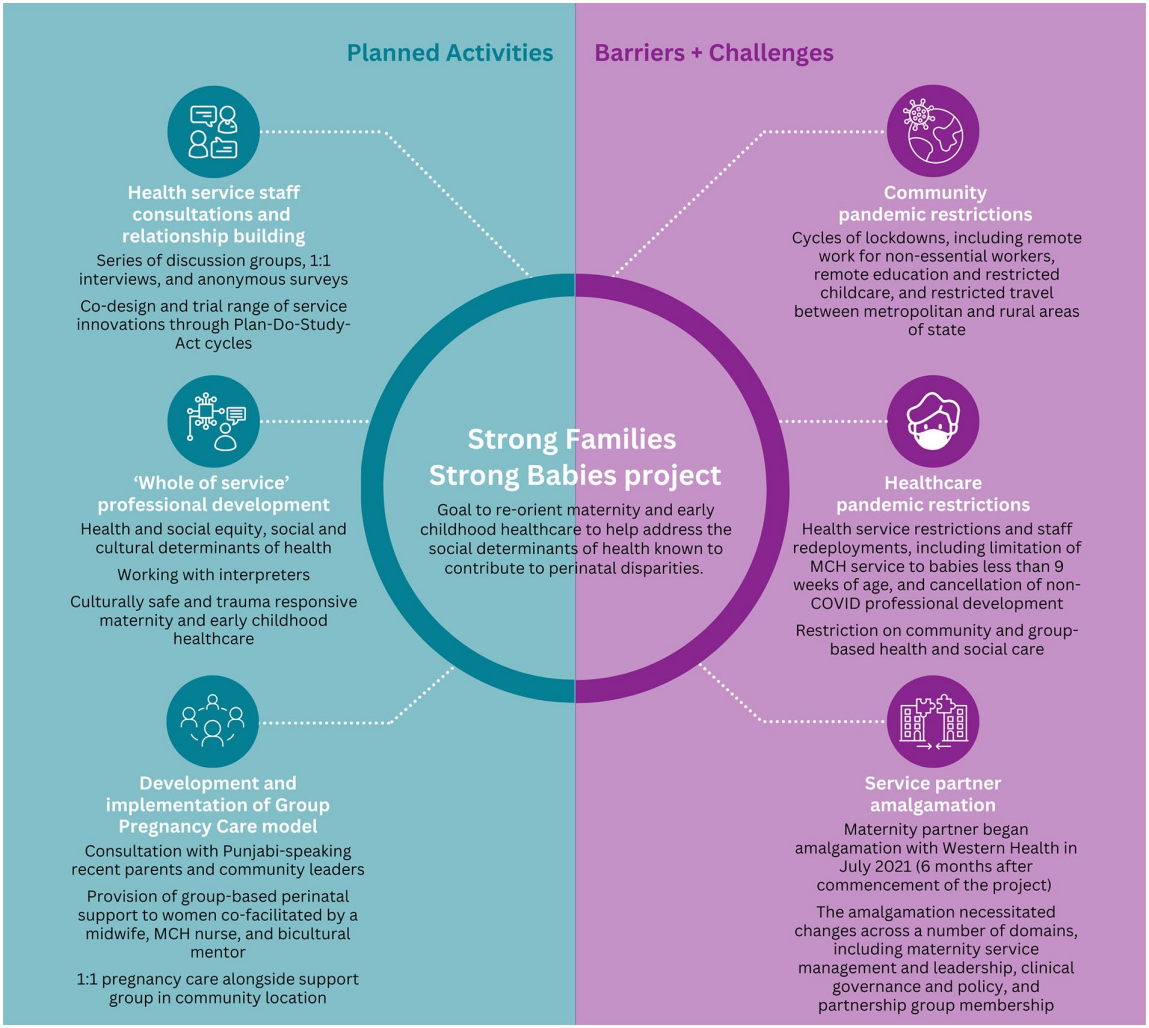
Project activities and challenges.

Project activities were undertaken during the height of the COVID-19 pandemic (January 2021-December 2022). Throughout this period, the state of Victoria was subject to stringent pandemic measures, including extended lockdowns and social distancing/gathering restrictions.^25-27^ The pandemic, an unforeseen hospital amalgamation, and longstanding workforce shortages prevented the partnership from implementing most planned project activities, including workforce consultation, professional development, and establishment of a new multi-disciplinary, collaborative model of Group Pregnancy Care^28^ for families of migrant background (Figure 1).

## Approach to Partnership Facilitation

We purposely adopted a model of collaborative practice to engage partner agencies in co-design of project activities from the beginning of the project. The model centres on agreed values and principles underpinning teamwork and collaboration, and includes a number of practical elements that support people to work well together in groups. More information about the model and its application within the project can be found in a separate evidence brief.^29^ The partnership group met regularly throughout the project; fortnightly in 2021, and monthly in 2022 with smaller working groups meeting to progress specific project objectives deemed feasible to implement in the context of pandemic restrictions and other constraints on services. Meetings were mostly held online, with longer face-to-face meetings scheduled when possible.

## Evaluation

An evaluation was conducted in early 2023 to understand what had and had not worked well, within the constraints affecting the partnership, and why, and to identify insights and recommendations to inform government and policy makers, health service managers, and teams seeking to undertake similar work in the future. Ten partnership members from 6 organisations participated in 1:1 interviews followed by a collaborative workshop designed to provide feedback on candidate themes and co-design recommendations.

Written consent was obtained from all participants prior to interviews and the workshop. Interviews were semi-structured, and audio recorded and transcribed. Detailed fieldnotes were generated in the event interview participants did not wish to be recorded, and also during the workshop. Data were analysed using reflexive thematic analysis; themes are structured around a central organising concept and reflect shared patterns of meaning across data items (Table 2).^30-32^ Illustrative quotes from partnership group members are provided, without attribution to protect confidentiality. Ethics approval was obtained from the Royal Children’s Hospital and Western Health Research Ethics Committees.

**Table 2.** Themes and Organising Concepts.

Theme	Central organising concept
There’s more than a pandemic standing in our way	Mapping realities of what really gets in the way of equity-oriented maternity and early childhood healthcare
We were invited to bring our whole selves	What it meant for us to be part of a team doing this work together
Despite everything, there are glimmers of hope for the future	How we understand the future of our fields and the equity agenda

## What We Learnt

### There’s More Than a Pandemic Standing in Our Way

COVID-19 measures had significant impacts on the group’s ability to co-design and implement practice change initiatives as planned. However, we understand the pandemic’s biggest impact to be in the way it powerfully amplified long-standing professional, cultural, political, and organisational factors that create significant barriers to increased focus on equity within maternity and early childhood healthcare. These factors include challenges in the way care is designed and funded, risk-focused priorities of large acute care services delivering maternity care, and long-standing issues with workforce wellbeing and burnout. The stressors and rapid unpredictable cycles of change necessitated by the pandemic occurred on top of these already challenged foundations, contributing to an environment characterised by precarity and scarcity, where trust and innovation can feel impossible for over stretched health and social care workforces. One partnership member reflected:

They’ve had three years of such unpredictability and such change at short notice that it’s hard to trust or believe in anything. . . we’re walking on fragile ground.

### We Were Invited to Bring Our Whole Selves

As a partnership group, we valued the invitation to bring ourselves as individuals, not just professionals, into the partnership team. As a team our strengths included our diverse personal and professional experiences and perspectives, demonstrating the value of working across agencies and professional groups. The investment of time and commitment to ongoing processes that enabled the formation and maintenance of shared purpose and connection was critical to our success. This shared purpose and connection fostered energy and commitment to enable us to navigate some of the more challenging experiences within the project, for example when COVID lockdowns necessitated significant changes to project plans. Members of the partnership group reflected on the importance and positive impact of these relationships:

I think if you love the people you’re working with, and you have a common goal, even though the outcome might not be what you think or what you anticipate, it’s still okay, there’s still learning in it.

It brings me huge joy that [this project] was a positive thing when absolutely everything in COVID was just so bloody hard.

### Despite Everything, There are Glimmers of Hope for the Future

Even after the myriad of challenges experienced within the life of this project, we still feel there is significant hope for future work seeking to enable equity-oriented maternity and early childhood healthcare. This reflects both our commitment to undertaking this work, and our capacity to see that the project was able to generate meaningful insights to inform future endeavours. We have co-designed our recommendations with this in mind, seeking to maximise the utility of our experiences and insights to help shape future work. Partnership members shared their perspectives on the future:

I can see the potential, even though it hasn’t quite worked, I can see how it could work. It gives me hope. . .

I think [the project] opened my mind to the possibilities of what’s out there and what can be done. I look at things now from different perspectives. . . I was looking from one side, but now maybe I can go around and have a look at other side too.

## Recommendations from the Partnership Group

The following recommendations have been co-created by the partnership group.

### Recommendations to Policy, Government, Service Managers

The partnership recognised there is an urgent need to invest in the prevention of health disparities. Without meaningful and sustained investment, cycles of intergenerational trauma and social inequity will not only persist, but they may also worsen. Meaningful investment includes:

1. Building project funding models and timelines that allow for meaningful engagement with communities and other key stakeholders, with attached accountabilities related to project objectives
2. Enabling senior managers to understand and support what is required to achieve equity, including investment of staff time and advocacy from those in leadership and executive positions
3. Enabling senior managers and those involved in making decisions about service design, funding and management to understand the economic and social benefits of preventing health disparities
4. Supporting multi-agency collaborations to share knowledge and skills, and work in partnership to promote equity

### Recommendations for Teams Undertaking Similar Work

The wellbeing of teams undertaking work of this kind is crucial to project sustainability and success. Team wellbeing can be supported and enhanced by:

1. Embedding purposeful, carefully planned group facilitation processes supporting teams to develop a sense of shared purpose, a culture of belonging, and the capacity to navigate challenges collaboratively
2. Taking the time to establish and maintain connections across organisations and between team members
3. Having representatives from the target group/s (eg, stakeholders, community members) embedded within the project as early as possible
4. Securing support from managers to allow team members to be properly involved, for example, attending partnership meetings, professional development, or reflective practice sessions

## Conclusion

Understanding how to form and sustain positive equity-focused multi-disciplinary multi-agency partnerships is critical to efforts to address health disparities. Whilst the COVID-19 pandemic created barriers to the accomplishment of project aspirations, our experiences of forming and sustaining a positive partnership throughout this period offer valuable insights for policy makers and services seeking to engage senior managers and practitioners in co-design and implementation of systems reform, and for workforces at the forefront of organisational change strategies required to promote health equity.
